# 

**Published:** 1856

**Authors:** 


### DENTAL REPORTER:

### AN INDEPENDENT JOURNAL,

DEVOTED TO

### DENTAL PROGRESS

AND

IMPROVEMENTS IN THE MANUFACTURE AND USE OF INSTRUMENTS AND MATERIALS.

COMPILED BY JNO. T. TOLAND.

ISSUED QUARTERLY.

S.G.O. 14 LIBRARY

PUBLISHED BY
TOLAND & THORNE,
WHOLESALE DEALERS IN TEETH, FOIL AND PLATE, INSTRUMENTS, FILES AND TOOLS, AND EVERY DESCRIPTION OF DENTIST'S MATERIALS,
No. 38 WEST FOURTH STREET, CINCINNATI, O.
1856—59.

